# Sub-therapeutic nevirapine concentration during antiretroviral treatment initiation among children living with HIV: Implications for therapeutic drug monitoring

**DOI:** 10.1371/journal.pone.0183080

**Published:** 2017-08-21

**Authors:** Bindu Parachalil Gopalan, Kayur Mehta, Reena R. D'souza, Niharika Rajnala, Hemanth Kumar A. K., Geetha Ramachandran, Anita Shet

**Affiliations:** 1 Division of Infectious Diseases, St. John’s Research Institute, St. John’s National Academy of Health Sciences, Bangalore, Karnataka, India; 2 The Institute Of Trans-Disciplinary Health Sciences and Technology (TDU), Bangalore, Karnataka, India; 3 Department of Pediatrics, St. John's Medical College Hospital, Bangalore, Karnataka, India; 4 National Institute for Research in Tuberculosis (Indian Council of Medical Research), Chennai, Tamil Nadu, India; Hôpital Bichat-Claude Bernard, FRANCE

## Abstract

Nevirapine, a component of antiretroviral therapy (ART) in resource-limited settings, known for auto-induction of metabolism, is initiated at half therapeutic dose until day 14 (‘lead-in period’), and subsequently escalated to full dose. However, studies have shown that this dosing strategy based on adult studies may not be appropriate in children, given that younger children have higher drug clearance rates. In this prospective cohort study, we studied trough plasma nevirapine levels by high performance liquid chromatography (HPLC) at days 7, 14 (lead-in period) and 28 (full dose period) after ART initiation amongst HIV-1 infected children initiating nevirapine-based ART in southern India. Among the 20 children (50% male, median age 9 years) included in the study, sub-therapeutic trough plasma nevirapine concentration (<4μg/ml) was seen in 65% (13/20) of children during the lead-in period within two weeks of ART initiation and among 10% of children at 4 weeks during full-dose nevirapine. Adherence was documented as ≥95% in all children by both caregiver self-report and pill count. Median nevirapine concentrations achieved at week 1 was 4.8 μg/ml, significantly lower than 8 μg/ml, the concentration achieved at week 4 (p = 0.034). Virological failure at one year of ART was observed in six children, and was not associated with median nevirapine concentration achieved during week 1, 2 or 4. We conclude that the dose escalation strategy currently practiced among young children living with HIV-1 resulted in significant subtherapeutic nevirapine concentration (≤4μg/ml) during the lead-in period. We call for a closer look at pediatric-focused dosing strategies for nevirapine initiation in young children. Further studies to establish age-appropriate threshold nevirapine concentration are warranted in young children to corroborate the role of therapeutic drug monitoring in predicting virological outcome.

## Introduction

Nevirapine was the first non-nucleoside reverse transcriptase inhibitor (NNRTI) approved by the FDA for pediatric use since 1998. Although nevirapine has a low genetic barrier to resistance, its triple advantages of virologic efficiency, safety and reasonable cost made it the most preferred NNRTI for paediatric use in resource limited settings [1]. The availability of fixed dose drug combination (FDC) tablets of zidovudine, lamivudine and nevirapine further simplified and cemented first-line treatment of pediatric HIV.

Notwithstanding its many advantages, nevirapine had pharmacokinetic aspects that merited a closer look, and set it apart from other antiretroviral drugs. Nevirapine is metabolized by the hepatic microsomal cytochrome P450 enzyme complex including CYP3A4/5 and CYP2B6, functioning both as a substrate and an inducer of cytochrome P450. Auto-induction of nevirapine metabolism generally sets in only by the second to fourth week of treatment initiation, resulting in a 1.5 to 2-fold increase in oral clearance. This delayed auto induction can give rise to potentially higher than expected plasma concentrations of nevirapine within the first two weeks [2]. To overcome the problem of higher concentration of nevirapine in blood and a subsequent greater probability of drug toxicities, a dose escalation strategy was used, where nevirapine was initiated at a half-therapeutic single daily dose during the first two weeks when metabolism is slower than usual, (the ‘lead-in period’), and after 14 days of therapy, the dose was escalated to the age-appropriate full therapeutic dose administered twice daily [3]. While this strategy appeared to solve the theoretical problem of high plasma drug concentration in the first two weeks of nevirapine initiation, the possibility of sub-therapeutic concentration due to higher nevirapine metabolism in young children was not taken into account [4]. Sub-therapeutic drug concentration during early therapy can result in slower viral clearance, increased risk of development of drug resistance and virological failure. Further, studies indicate that nevirapine metabolism in young children aged ≤8 years was more rapid than that observed in children >8 years, and that younger children required higher doses of nevirapine to achieve therapeutic concentration [5–7].

Studies conducted among adult cohorts indicated the risk of greater drug toxicity when this dose escalation strategy was not used, although a consistent relationship between toxicity and nevirapine pharmacokinetic parameters has not been observed [8,9]. A randomized controlled trial in Zambia comparing full-dose nevirapine versus a dose escalation strategy among children, did not reveal higher degree of toxicity among those children who received full dose nevirapine at initiation [10]. Thus, virological failure due to underexposure and possibly lower than optimal dosing is of greater concern than toxicity due to overdosing in young children [11,12].

Maintaining optimal plasma concentration of nevirapine is critical, because a single point mutation at specific position on the *pol* gene on the HIV-1 genome may confer high-level resistance to nevirapine [13,14]. Studies have indicated that trough plasma nevirapine concentration (C_*trough*_) of </ = 3μg/ml is associated with increased risk of treatment failure by promoting the evolution of drug resistant viral variants [15]. Data largely from adult studies were conflicting, suggesting a range of target nevirapine concentrations from 3 to 4.3 μg/ml [16–19]. Additionally, there is no consensus regarding the target drug concentration to be achieved for successful virological control.

Very few studies have explored the pharmacokinetics of nevirapine in children, particularly in the early period of ART initiation. We undertook this study aiming to describe nevirapine pharmacokinetics in HIV-infected young Indian children who were initiated on nevirapine containing ART that used a dose escalation strategy for nevirapine. We also aimed to evaluate the impact of sub-therapeutic nevirapine concentration during dose escalation and steady state on early immunological and virological outcomes.

## Materials and methods

### Patient population and study design

This longitudinal cohort study was conducted from January 2013 to February 2015 at the Infectious Disease clinic at St. John’s Medical College Hospital, Bangalore. Following approval of the study by the Institutional Review Board, written informed consent was obtained from parents or caregivers, and assent was obtained from children older than 7 years of age who were eligible and willing to participate in the study. Eligible children were HIV-1 positive, aged between 2–18 years and initiating nevirapine-containing ART regimen. Children with serious life-threatening illnesses, a reported history of maternal antenatal NNRTI exposure, or those with elevated liver enzymes at baseline were excluded from the study.

At enrolment, baseline history, anthropometry and physical examination were documented. Routine laboratory tests performed at baseline included complete blood count, alanine transaminase (ALT), aspartate transaminase (AST), and CD4+ T cell count. Blood samples were obtained for the evaluation of HIV-1 RNA load and drug resistance profile at baseline (week 0) prior to ART initiation. Nevirapine was initiated at half-therapeutic dose (120–150 mg/m^2^ body surface area (BSA)/dose once a day up to a maximum dose of 200 mg once a day) along with full-dose zidovudine and lamivudine based on WHO weight band dosing criteria as recommended by the National AIDS Control Organization Paediatric ART guidelines [20]. A fixed dose combination (FDC) of zidovudine, lamivudine and nevirapine was administered in the morning followed by FDC of zidovudine and lamivudine in the evening. At the end of week 2 (Day 15), after the lead-in dosing period, the dose of nevirapine was escalated to full dose (120–150 mg/m^2^ BSA/dose twice daily up to a maximum dose of 200 mg twice daily) without a change in the other two medications (FDC of zidovudine, lamivudine and nevirapine twice daily). Children were followed closely for one year, with scheduled study visits at weeks 1, 2, 4, 8, 12, 24 and 48. At each study visit adherence to therapy was carefully reviewed, by pill count and self-report by the child or caregiver. Pill count was conducted by trained staff during the clinic visit, and adherence computed by dividing the number of pills presumably consumed during the period between visits, by the number of pills dispensed for that time period multiplied by 100 [21]. Average adherence (based on the average of pill count and self-report) was categorized as >95% adherence, 75 to 95% adherence and <75% adherence [12].

### Laboratory analysis

Six ml of whole blood was collected in an EDTA vacutainer tube during each study visit before ART administration for measuring trough plasma nevirapine level, HIV-1 viral load and drug resistance genotyping. Plasma was separated out by centrifuging the whole blood at 1500 x g for 15 minutes at 4°C, aliquoted in 1 ml screw capped vials and stored at -80°C until analysis.

CD4 cell counts were obtained by flow cytometry (Becton Dickinson, CA, USA) at baseline, week 24 and week 48. HIV-1 RNA viral loads were evaluated in the stored plasma samples using Abbott Real Time HIV-1 assay (Abbott Molecular Inc., Des Plaines, IL, USA) at all study visits. Trough plasma nevirapine concentration were estimated at the end of week 1 and week 2 (lead-in period) and week 4 (steady-state period) of ART initiation.

Nevirapine pharmacokinetic testing was performed at National Institute for Research in Tuberculosis, Chennai, India by a validated HPLC method (Shimadzu Corporation, Kyoto, Japan) with UV detection using 3-Isobutyl-1-methyl xanthine as internal standard for the assay [22]. Nevirapine concentrations ranging from 0.5 to 10 μg/ ml were used for the preparation of standard curve which was found to be linear. The lowest detection limit of the assay was 0.25 μg/ml. Repeatability studies were performed for quality control; 10% of samples were re-analysed in a blinded manner, and the percentage variation was below 10%. A plasma nevirapine level of 4 μg/ml was chosen as the target trough plasma nevirapine level to be achieved for long term virological control for the purpose of this study in line with two other studies that compared nevirapine concentration and virological outcome (3.9 μg/ml [18], 4.3 μg/ml [19]). Therapeutic response was assessed by immunological and virological outcome at week 48 of therapy. Immunological failure was defined as persistent CD4 count below 200 cells/mm^3^ or CD4 percentage <10%. Virological failure at one year of ART was defined as the detection of HIV-1 RNA viral load ≥200 copies/ml [23].

Genotypic HIV Drug resistance (HIVDR) testing was performed retrospectively by population sequencing (Sanger method) in stored plasma samples at baseline and at week 48 if viral loads were ≥1000 copies/ml. One ml plasma was centrifuged at 20000 x g for 1 hour at 4°C to concentrate the virus and RNA was extracted using Qiagen QIAmp viral RNA mini kit following manufacturer’s protocol (QIAamp viral RNA extraction kit, Germany). First strand cDNA synthesis was performed using revert aid reverse transcriptase enzyme (Thermo scientific, MA, USA) and random hexamers (Promega). Nested PCR for HIV-1 *pol* which comprises of Reverse Transcriptase amino acid 17 to 235 (HxB2 position 2598 to 3250) region where majority of drug resistance mutations (DRMs) tend to get accumulated was performed as described previously [24]. The products (size 652 bp) were gel purified using QIAquick Gel extraction kit and sequenced in an ABI PRISM® 310 Genetic Analyzer (Applied Biosystems, CA, USA). Drug resistance mutations were analysed by Stanford University Genotypic Resistance Interpretation Algorithm (HIVdb version 8.2) [25]. A maximum likelihood phylogenetic tree was constructed (MEGA 6.0) from the generated sequences along with sub type specific reference sequences downloaded from Los Alamos HIV data base (http://www.hiv.lanl.gov/.) for determining HIV-1 subtypes. Viral RNA sequences analysed in this study have been submitted to GenBank with accession numbers MF359600 to MF359625.

### Statistical analysis

All statistical analyses were performed using SPSS 24.0 statistical package. Categorical variables describing patient characteristics were described as frequencies (%) and median (with interquartile ranges at 25^th^ and 75^th^, IQR). Difference between trough plasma nevirapine concentration during the lead-in period of weeks 1 and 2, and steady state period (week 4) were assessed by Wilcoxon signed rank test. Spearman’s correlation coefficient was used to test the association between outcome variables (weekly nevirapine levels and viral load). Mann-Whitney U test was used when two groups were compared (plasma nevirapine concentration between groups of virological failures and viremia controllers). For all tests, p <0.05 was considered to be statistically significant.

## Results and discussion

### Results

A total of 31 children who initiated nevirapine-containing ART regimen were screened at the study site during 2013. Of them, 28 consented for participation in this study. During the observation period, six children experienced ART interruption and had to drop out of the study (four developed nevirapine-induced toxicity and two developed adverse events related to zidovudine and cotrimoxazole). Further, two children were transferred to another centre, resulting in a final cohort of 20 children (median age 9 years; males 50%) with complete data for 48 weeks. None of these children experienced ART or cotrimoxazole prophylaxis related adverse events. Demography and laboratory parameters of the included children are presented in Table 1.

**Table 1:** Characteristics of children included in the study (n = 20).

		
	Week 0	Week 48
Age (years)—median (IQR)	9 (6, 11)	
Male n (%)	10 (50)	
CD4%—median (IQR)	17 (12.5, 23.5)	31 (26.7, 36.5)
HIV-1 RNA log_10_ copies/ml Median (IQR)	5.4 (5.0, 6.0)	3.9 (3.7, 4.7)
Adherence (%)	>95	>95
Drug resistance detected—n (%)	1 (5)	5 (25)

IQR: Interquartile range

All children reported >95% adherence to therapy at each clinic visit throughout the study period. Phylogenetic analysis of the *pol* sequence derived from plasma virus at baseline revealed that all children were infected with HIV-1 subtype C. At week 48, virological failure was observed in 30% (6/20) of children. Immunological failure was not observed as the median CD4 percentage rose from 17 (IQR 12.5, 23.5) at baseline to 31 (IQR 26.7, 36.5) at week 48. Median CD4% of those in virological failure was 30% (IQR 25, 35).

**Fig 1:**
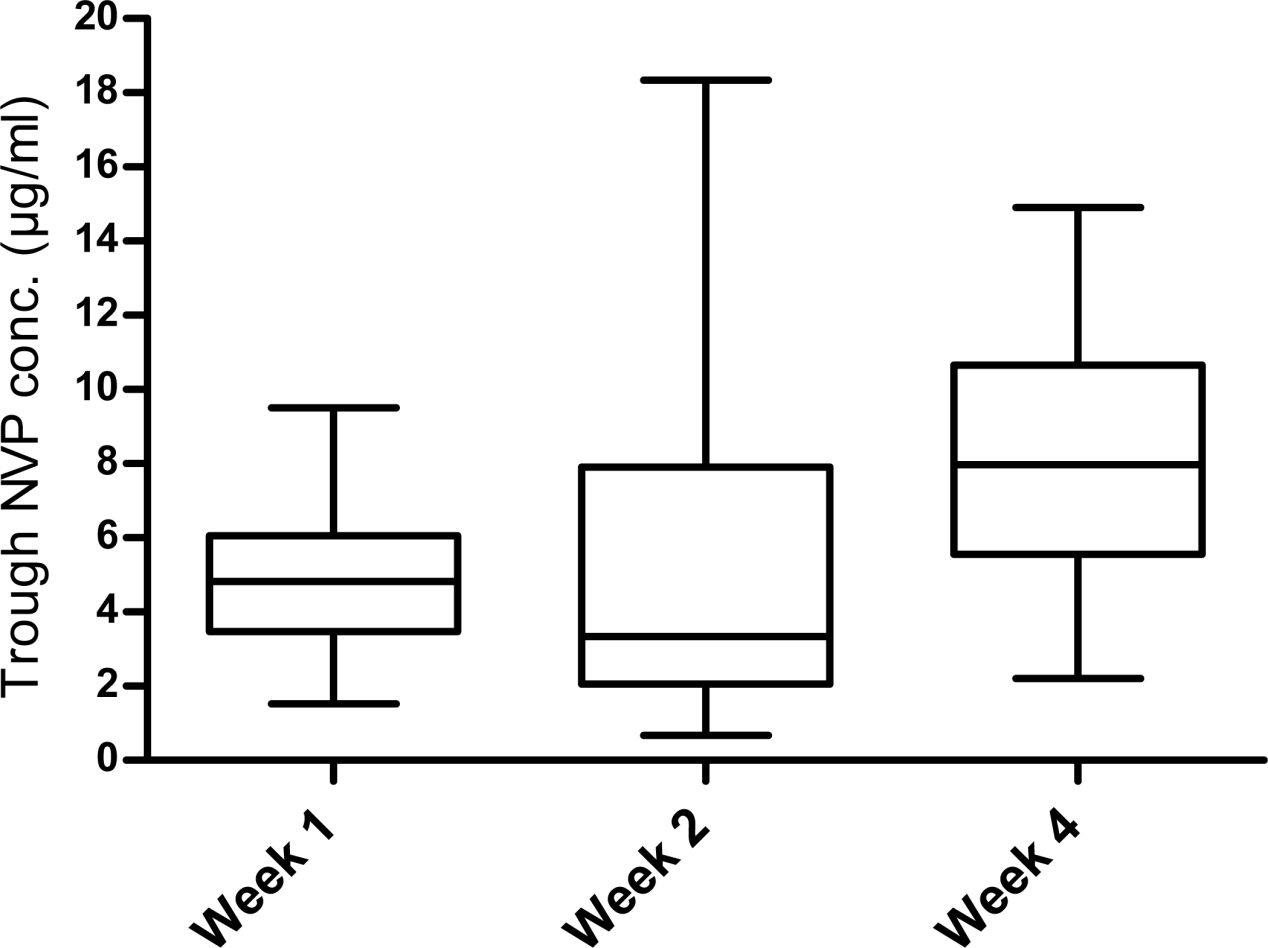
Comparison of trough plasma nevirapine concentration (μg/ml) during lead-in and steady state period. The difference between lead in (week1, 2) and steady state (week4) trough plasma nevirapine concentration (p = 0.034, p = 0.132) were assessed by Wilcoxon signed rank test.

During the lead-in period, sub-therapeutic trough plasma nevirapine concentration (≤4μg/ml) was seen in 65% (13/20) of children initiating ART. This included 40% (8/20) at week 1, and 55% (11/20) at week 2 of ART initiation. After dose escalation, sub-therapeutic concentration was seen in 10% (2/20) of children at week 4. Median trough plasma nevirapine concentration during the lead-in period was 4.8 μg/ml (IQR 3.5, 6.1) at week 1 and 3.4 μg/ml (IQR 2.1, 7.9) at week 2. The steady state median trough nevirapine concentration measured at week 4 was 8 μg/ml (IQR 5.6, 10.7). Median nevirapine concentration achieved at week 1 was significantly lower than the concentration achieved at week 4 (Wilcoxon signed rank; p = 0.034,) although statistically significant difference was not observed between week 2 and week 4 (p = 0.132). (Fig 1).

There were six children who were in virological failure at week 48. No significant correlation was observed between plasma nevirapine concentration at week 1, 2 or 4 and viral load achieved at week 48 (Spearman’s rho; p ≥0.05). During the lead-in period, 66.6% (4/6) of virological failures and 64.2% (9/14) of viremia controllers had subtherapeutic nevirapine concentration. Median nevirapine concentration observed among the two groups were 4.8 μg/ml (IQR 3.4, 8.8) vs. 4.8 μg/ml (IQR 3.5, 6.1) at week 1, 7.1 μg/ml (IQR 1.9, 11.5) vs. 3.1 μg/ml; (IQR 2.1, 7.7) at week 2, 6.1 μg/ml(IQR 4.1, 8.5) vs. 8.4 μg/ml (IQR 5.5, 11.5) at week 4. Although there was no significant difference in the median nevirapine concentration achieved at week 1, 2 or 4 between virological failures and viremia controllers (Mann-Whitney U test, p = 0.967, p = 0.433, p = 0.187 respectively) baseline CD4% differed significantly between the two groups (p = 0.047) (Table 2).

**Table 2:** Variables of observed among virological failures and viremia controllers.

	Virological failures	Viremia controllers	
	≥200 copies/ml	<200 copies/ml	
	(n = 6)	(n = 14)	p
**Age (years)—median (IQR)**	7.5 (6, 7)	9.5 (6, 11)	0.156
**CD4%—median (IQR)**			
Baseline	14 (10, 16)	21 (15, 26)	0.047
Week 48	30 (26, 35)	31.5 (30, 40)	0.343
**HIV-1 RNA log**_**10**_** copies/ml—median (IQR)**	** **	** **	
Baseline	5.9 (5.5, 6.1)	5.3 (5.0, 5.6)	0.117
Week 1	4.0 (3.4, 4.5)	3.6 (3.2, 3.8)	0.083
Week 2	3.5 (3.0, 4.0)	3.1 (2.7, 3.3)	0.107
Week 4	3.5 (2.8, 3.8)	2.8 (2.2, 3.2)	0.039
**C**_***trough***_ **NVP conc. μg/ml- median (IQR)**			
Week 1	4.8 (3.4, 8.8)	4.8 (3.5, 6.1)	0.967
Week 2	7.1 (1.9, 11.5)	3.1 (2.1, 7.7)	0.433
Week 4	6.1 (4.1, 8.5)	8.4 (5.5, 11.5)	0.187
**Drug resistance detected- n (%)**			
Baseline	1 (16.6)	0	
Week 48	5 (83.3)	0	

The two groups, virological failures (HIV RNA ≥200 copies/ml) and viremia controllers. (<200 copies/ml) were compared with Mann-Whitney U test. P <0.05 was considered to be statistically significant.

Among those children who experienced virological failure, one child had nevirapine associated resistance mutations K101E and E138G observed at baseline and at virological failure. Newly acquired drug resistance mutations were observed in 4 children at virological failure, and among them 3 had subtherapeutic nevirapine levels during the lead-in period. The mutations associated with nevirapine resistance observed were K103N (n = 2), G190A (n = 2), Y181C (n = 1), V108I (n = 1), K101E (n = 1), and E138G (n = 1). NRTI resistance mutations M184V and V75I were also observed. Baseline drug resistance was not observed among the viremia controllers.

Table 3 displays the median trough plasma nevirapine concentration and therapeutic response as a function of age. Subtherapeutic nevirapine concentration were seen in 87.5% (7/8) of children ≤8 years, and in 50% (6/12) among children >8 years. Lead-in (week1) and steady state nevirapine concentration was significantly different between the age groups (p = 0.023, p = 0.011). The proportion of children experiencing virological failure was higher in the ≤8 years age group (50%) in comparison to the >8 years age group (16.6%) although there was no statistical significance (Mann Whitney U p = 0.16).

**Table 3:** Nevirapine pharmacokinetics and therapeutic response based on age.

	≤8 Years (n = 8)	>8 Years (n = 12)	p
C_*trough*_ nevirapine concentration (μg/ml) median (IQR)	** **		
Week 01	3.7 (2.5, 4.8)	5.9 (4.2, 7.2)	0.023
Week 02	3.3 (1.2, 7.4)	4.4 (2.4, 9.7)	0.418
Week 04	5.6 (3.9, 7.5)	8.7 (7.4, 11.9)	0.011
Nevirapine concentration ≤ 4μg/ml during lead-in period -n (%)	7 (87.5)	6 (50)	
Virological failure at week 48—n (%)	4 (50)	2 (16.6)	0.16
Resistance detected at virological failure—n (%)	4 (50)	1 (8.3)	

## Discussion

In this study, we found that ART initiation in young children using the dose escalation strategy for nevirapine resulted in significant sub-therapeutic nevirapine concentration during the lead-in period compared to the steady state period. Sub-therapeutic nevirapine concentration were more pronounced in children ≤8 years of age; supporting earlier observations that younger children metabolized nevirapine more rapidly than older children [4,5,7].

Previous studies on pharmacokinetics in adults have indicated slow induction of nevirapine metabolism with initial doses, while later doses were associated with faster metabolism [26]. The strategy of dose-escalation for nevirapine was largely derived from studies conducted in adult cohorts [27,28]. However, the relevance of extrapolating adult data to the pediatric population has been a matter of considerable debate. Previous studies on nevirapine pharmacokinetics in children demonstrated that nevirapine clearance increased with decreasing age, and children younger than 8 years have higher oral clearance when compared to older children, suggesting the requirement of higher doses to achieve equivalent drug exposure in these young children [5]. However, nevirapine pharmacokinetic data combined from eight pediatric clinical trials representing a total of 639 subjects from multiple continents and across the pediatric age continuum demonstrated that the age effect on clearence was modest and found that clearence increased with age in the first year of life [29]. Sub-therapeutic nevirapine concentration during the lead-in period have been reported from various adult studies as well [30,31]. The CHAPAS-1 trial that included 79 children (>2yrs) on nevirapine dose escalation reported sub-therapeutic nevirapine concentration in 12% children, and found no association between virological failure and plasma drug concentration at week 2 [1]. Subtherapeutic nevirapine concentration attributable to rapid drug metabolism in children <8 years has also been reported by Ellis et al [4]. In keeping with existing literature, our findings suggest that dose escalation strategy resulted in suboptimal drug concentration and may not be appropriate for young Indian children, especially for those younger than 8 years of age.

The minimum threshold drug concentration required for therapeutic drug monitoring varies widely across various studies ranging from 3 to 4.3 μg/ml. Veldkamp *et al* suggested a target concentration of 3.4 μg/ml from 12 week onwards for controlling viral loads to <50 copies/ml in adults on dose escalation [17]. Wang *et al* recommended a target concentration of 3.9 μg/ml for therapeutic drug monitoring in Chinese adult population [18]. Requena *et al* suggested a concentration of >4.3 μg/ml for the complete suppression of all polymorphic natural variants [19]. Studies conducted in children by Fillekes *et al* (CHAPAS trial), Ellis *et al*, Zoufaly *et al*, Nikanjam *et al*, Corbet *et al* and Vanprapar *et al* had chosen nevirapine concentration of <3 μg/ml as subtherapeutic [1,4,12,29,32,33] while Ramachandran *et al*, Chokephaibulkit *et al* and Mukherjee *et al*, had chosen a minimum trough level of 3.4 μg/ml [34–36]. These threshold nevirapine concentrations were established largely from adult studies and did not explore drug pharmacokinetics during dose escalation, and thus may not be applicable for young children, especially in the setting of higher apparent oral clearance as described earlier. We used a cut off value of 4 μg/ml, and still found a substantial proportion of children with sub-therapeutic drug concentration during the lead-in period. Although the steady state nevirapine concentration achieved was in therapeutic range in a majority of children, the extremely low levels observed during week 1 and 2 may predispose to the selection of resistance and virological failure.

A number of studies have reported an association between subtherapeutic plasma nevirapine concentration and poor virological outcome [12,15,17,19]. However, few other studies have found lack of association ([1,34,37,38] thereby questioning the role of therapeutic drug monitoring to predict virological outcome. Requena *et al* found a therapeutic trough nevirapine concentration window to predict longer virological suppression [19], while Kimulwo *et al* [39] associated the increase in early peak nevirapine concentration at 4 hours of ART to lower viral loads. Thus, so far there is no consensus opinion on what might be the ideal target drug levels, time point for evaluating drug concentration, and peak/trough levels to be evaluated in predicting virological outcome.

In this study, we failed to observe a clinically relevant effect of nevirapine concentration on virological outcome. This finding could have been affected by various factors, mainly the small sample size and possible role of primary or acquired drug resistance among minor viral variants. Recruitment was halted after we noted high level of nevirapine-based adverse reactions in several of the enrolled children. The national policy change in the choice of first-line NNRTI treatment regimen from nevirapine to efavirenz/atazanavir during the course of the study precluded further recruitment for the study. Additionally, virological failure in a therapy adherent patient can be attributable to drug resistant minor viral variants that were pre-existing or evolved during the course of therapy [40]. In an ART regimen containing a low genetic barrier drug like nevirapine, viral variants carrying single point mutations (K103N or Y181C) evolve rapidly irrespective of plasma concentration of the drug.

The larger interpretation of this study needs to consider some potential limitations. The major limitations of this study are the small sample size and the lack of information about the pharmacogenetic determinants of plasma nevirapine concentration. Single nucleotide polymorphism (SNPs), in the gene encoding drug metabolizing liver enzyme cytochrome P450 2B6 (CYP2B6) has well known impact on plasma nevirapine levels [41,42]. SNPs at exon 4 (516 G > T) and exon 7 (983 T > C) of CYP2B6 are known to reduce the catalytic activity of enzyme resulting in higher trough plasma drug levels [41,43] suggesting that lead in dosing could be more appropriate for a 516TT or 983CC genotype individual. In view of the finding by Varshney *et al* that approximately 20% of the Indian population are poor drug metabolizers [44], there is an increased likelihood of a rapid metabolizer genotype 516 GG or GT in our cohort which might have confounded our results. Nevertheless, this is the first longitudinal study in India that assessed nevirapine concentration in children during the dose escalation period at ART initiation, thus contributing important information on nevirapine pharmacokinetics in children.

Although nevirapine-based ART is not the preferred first-line ART regimen currently, it still remains an effective alternative first-line ART regimen for children, adolescents and adults [45]. As the principally recommended stand-alone formulations and fixed drug combinations of dolutegravir and efavirenz are not widely available and affordable especially in low and middle income settings, the WHO recommends the use of nevirapine-based regimen as alternate first-line regimen in these settings [45]. Nevirapine also remains the NNRTI of choice for newborns initiating treatment at birth, and an alternative option when clinically required by patients who cannot tolerate or have contraindications to other NNRTIs. Thus, our finding of sub-therapeutic concentration during lead-in period and subsequent resistance acquired early in the course of nevirapine therapy, although not statistically significant, holds relevance and can impact the future available therapeutic options in children and adolescents living with HIV.

We conclude that the dose escalation strategy practised in HIV-1 infected young children initiated on nevirapine-containing ART in India resulted in a significant proportion of children with sub-therapeutic nevirapine concentrations during the lead-in period. This study finding has important clinical implications considering the continued use of nevirapine as an alternate option in the first-line ART regimen in children. Our findings underscore the need for a thorough review of the nevirapine dose escalation strategy in children. Further studies conducted in larger cohorts are warranted for establishing age-appropriate threshold nevirapine concentration in young children and to corroborate the role of therapeutic drug monitoring in predicting virological outcome.

## Supporting information

S1 Table Weekly nevirapine levels, viral loads and drug resistance mutations detected in the cohort. NA: Not applicable, NVP: nevirapine, DRMs; Drug resistance mutations. C_*trough*_ NVP Conc.: Trough plasma nevirapine concentration.
(PDF)
