# Supplementary material for: Sub-therapeutic nevirapine concentration during antiretroviral treatment initiation among children living with HIV: Implications for therapeutic drug monitoring
Source: PLoS One. 2017 Aug 21;12(8):e0183080. doi: 10.1371/journal.pone.0183080 (PMC5565187; doi:10.1371/journal.pone.0183080)
Supplement: S1 Table — NA: Not applicable, NVP: nevirapine, DRMs; Drug resistance mutations. Ctrough NVP Conc.: Trough plasma nevirapine concentration. (PDF) [file pone.0183080.s001.pdf]

S1 Table. Weekly nevirapine levels, viral loads and drug resistance mutations detected in the cohort.

Patient	Age	Sex	C_{trough} NVP Conc. µg/ml			Viral load log_{10} copies/mL			DRMs	
			Week 1	Week 2	Week 4	Week0	Week 24	Week 48	Baseline	Beyond Week 48
**NVP02**	13	M	3.44	3.07	11.2	4.96	2.18	1.69	None	NA
**NVP03**	9	F	7.25	7.69	5.56	4.94	1.69	1.69	None	NA
**NVP05**	8	F	5.78	16.03	4.39	6.01	2.32	3.77	None	**M184V-V108I, Y181C**
**NVP06**	7	F	3.37	7.69	3.37	5.71	3.75	3.79	None	**V75I, K103N**
**NVP08**	14	M	4.03	2.56	8.42	5.54	1.69	1.69	None	NA
**NVP10**	12	M	6.08	5.49	8.34	5.21	1.69	2.18	None	NA
**NVP12**	2	M	1.61	3.15	7.61	6.16	2.18	2.18	None	NA
**NVP13**	5	F	3.58	0.76	5.48	6.40	2.33	2.18	None	NA
**NVP14**	10	F	4.49	7.99	13.01	5.29	2.48	1.69	None	NA
**NVP15**	6	M	3.8	6.62	5.71	6.02	3.09	5.05	None	**M184V, K103N, G190A**
**NVP16**	9	M	9.51	1.98	6.47	4.71	1.69	3.39	None	**M184V, G190A**
**NVP17**	6	M	1.52	1.67	9.05	6.45	4.35	4.52	**K101E, E138G**	**K101E, E138G**
**NVP18**	9	F	6.92	2.28	14.91	5.49	1.69	1.69	None	NA
**NVP19**	11	M	6.01	14.08	8.9	4.76	1.69	1.69	None	NA
**NVP20**	10	M	5.17	3.2	11.34	5.17	2.18	2.18	None	NA
**NVP23**	9	F	8.67	11.49	8.37	5.88	2.49	4.09	**None**	**None**
**NVP24**	11	M	2.05	1.52	5.55	4.10	1.69	1.69	None	NA
**NVP25**	6	F	6.01	0.68	2.21	5.23	1.69	1.69	None	NA
**NVP26**	9	F	5.86	18.34	12.1	5.49	1.69	1.69	None	NA
**NVP27**	6	F	3.8	3.5	7.23	5.89	2.18	1.69	None	NA
